# Wolf Creek XVIII Part 7: strategies to optimize international collaborations in cardiac arrest research^☆^

**DOI:** 10.1016/j.resplu.2026.101241

**Published:** 2026-01-26

**Authors:** Yohei Okada, Janet E. Bray, Robert W. Neumar, Bryan F. McNally, Markus B. Skrifvars, Laurie J. Morrison, Niklas Nielsen, Theresa Olasveengen, Marcus E.H. Ong

**Affiliations:** a Pre-hospital and Emergency Research Centre, Health Services Research and Population Health, Duke-NUS Medical School, Singapore, Singapore; b School of Public Health and Preventive Medicine, Monash University, Melbourne, Australia; c Pre-hospital, Resuscitation & Emergency Care Research Unit (PRECRU), Curtin University, Perth, Australia; d Department of Emergency Medicine and Department of Molecular & Integrative Physiology, University of Michigan Medical School, Ann Arbor, MI, USA; e Department of Emergency Medicine, Emory University School of Medicine, Atlanta, GA, USA; f Department of Anaesthesiology and Intensive Care Medicine, Helsinki University Hospital and University of Helsinki, Finland; g Division of Emergency Medicine, Department of Medicine, University of Toronto, Sunnybrook Health Sciences Centre, Toronto, Ontario, Canada; h Department of Clinical Sciences Lund, Anesthesiology and Intensive Care, Lund University, Lund and Department of Anesthesiology and Intensive Care, Helsingborg Hospital, Helsingborg, Sweden; i Institute for Clinical Medicine, University of Oslo and Department of Anesthesia and Intensive Care Medicine, Oslo University Hospital, Oslo, Norway; j Dept of Emergency Medicine, Singapore General Hospital, Singapore, Singapore

**Keywords:** Clinical trial, Registry, Preclinical research, Global collaboration

## Abstract

**Introduction:**

International collaborations in research for cardiac arrest are much needed to advance the science, translate this into practice and implement for impact. However, barriers and challenges remain for international collaboration. This paper aims to summarize the key discussions and consensus recommendations from the Wolf Creek XVIII Conference, focusing on strategies to optimize international collaborations in cardiac arrest research.

**Methods:**

The 50th Anniversary Wolf Creek XVIII Conference was hosted by the Max Harry Weil Institute for Critical Care Research and Innovation in Ann Arbor, Michigan, USA on June 19–21, 2025. Strategies to Optimize International Collaborations in Cardiac Arrest Research was a topic of focused presentations and discussions by invited panelists and conference participants, made up of international academic and industry scientists, as well as thought leaders in the field of cardiac arrest resuscitation. An expert panel gave perspectives and insights that were debated and feedback was given by participants.

**Results:**

Discussions were organized into three domains: registry research, basic science and translational research, and clinical trials. Several large-scale registries have collectively advanced data-driven resuscitation science through collaboration and mutual learning, while continuing to face challenges related to heterogeneity, privacy regulation, and data lag. Successful models like the Global Out-of-Hospital Cardiac Arrest Registries (GOHCAR) consortium highlight the importance of trust and sustained engagement. In preclinical research, the Transcontinental Cardiac Arrest Experimental Network for Discovery (TRANSCEND) aims to harmonize international laboratory studies. Clinical collaboration is progressing through multicenter randomized controlled trials such as the Sedation, temperature and pressure after cardiac arrest and resuscitation (STEPCARE), promoting inclusive, adaptive global research.

**Conclusion:**

Sustained international collaboration across registries, laboratory studies, and clinical trials is key for advancing resuscitation science. By fostering trust, harmonization, and capacity building, these global networks can accelerate discovery and improve outcomes across cardiac arrest and other time-critical conditions.

## Introduction

Cardiac arrest, whether it occurs as an out-of-hospital cardiac arrest (OHCA) or in-hospital cardiac arrest (IHCA), is a major global health challenge.^1^ Despite remarkable scientific progress, survival with favorable neurological outcomes is still low and differs greatly between and within regions.^2^ Resuscitation science is multidisciplinary, encompassing basic and translational research, pre-hospital and in-hospital care, critical care, rehabilitation, public health and ethics. Its progress relies on the integration of diverse fields, including medicine, physiology, psychology, data science, engineering, sociology and implementation research.^3^

Global collaboration enables researchers to leverage the complementary strengths of diverse health systems, patient populations, and approaches to care.^4^, ^5^ The International Liaison Committee on Resuscitation (ILCOR) has played a central role in fostering global collaboration, particularly in harmonizing scientific evidence evaluation, and promoting the dissemination of resuscitation guidelines worldwide. Within this research context, working together across continents allows investigators to examine variations in cardiac arrest epidemiology, emergency response systems, and post-resuscitation practices, thereby identifying both universal principles and context-specific solutions.^1^, ^4^, ^5^, ^6^, ^7^, ^8^ Large-scale multicenter data sharing provide the statistical power and external validity that single-country studies cannot achieve, ensuring that research findings are robust and generalizable.^9^

Collaboration across countries with differing resources also fosters mutual learning and innovation. High-resource settings can contribute advanced technologies, data infrastructure, and methodological expertise, while low- and middle-income countries (LMICs) offer invaluable insights into adaptability, scalability, and cost-effectiveness in real-world, resource-limited environments.^10^, ^11^ Such partnerships not only advance scientific discovery but also enhance global equity, ensuring that the benefits of resuscitation research extend beyond regional boundaries.

Even though global collaboration in resuscitation science has several benefits, translating the vision of global collaboration into practice remains challenging. To further advance international collaboration, it is essential to recognize these barriers and explore potential solutions toward achieving more effective and equitable global partnerships in resuscitation science.

The Wolf Creek XVIII Conference, marking the 50th anniversary of a long-standing tradition of intellectual exchange, provided a timely opportunity to reflect on the current state and future direction of global collaboration in cardiac arrest research. This paper summarizes the discussions from the session “Strategies to Optimize International Collaborations in Cardiac Arrest Research,” highlighting key barriers, enablers, and priorities for advancing global partnerships in resuscitation science.

## Methods

### The Wolf Creek Conference

Since its inception in 1975, the Wolf Creek Conference has established a tradition of providing a unique forum for robust intellectual exchange between thought leaders and scientists from academia and industry, with a shared goal of advancing the science and practice of cardiac arrest resuscitation.^12^ The 50th Anniversary Wolf Creek XVIII Conference was hosted by the Max Harry Weil Institute for Critical Care Research and Innovation in Ann Arbor, Michigan, USA, on June 19–21, 2025. Participants included international academic and industry scientists and thought leaders in cardiac arrest research. All attendees completed conflict-of-interest disclosures before the meeting.

### Discussion process

“Strategies to Optimize International Collaborations in Cardiac Arrest Research” was one of six focused panel sessions. Each included short presentations by invited experts followed by structured group discussions. The goal was to explore the current and future state of global cardiac arrest research, identify knowledge gaps, and define priorities for the next decade. Panels were composed of multidisciplinary experts with experience in international collaboration, multicenter data harmonization, and global research networks. Panelists first outlined key barriers and enablers, which were then debated and refined through open discussion to generate consensus recommendations.

All discussions were moderated and recorded to ensure accurate capture of key ideas and consensus points. The resulting synthesis of presentations and deliberations, including the most salient strategies to enhance international collaboration in cardiac arrest research, are presented and discussed in this manuscript.

## Results

### Current state of international collaborations in cardiac arrest research

#### Registries

International cardiac arrest registries have become the backbone of collaborative research, providing a foundation for benchmarking, comparative analyses, and system improvement across regions.^9^ Over the past two decades, major collaborative networks have transformed the landscape of data-driven resuscitation science using a standardized data collection format.^4^, ^5^ Prominent initiatives such as the Cardiac Arrest Registry to Enhance Survival (CARES) in North America, the Pan-Asian Resuscitation Outcomes Study (PAROS), the Aus-ROC in Australia, and the European Registry of Cardiac Arrest (EuReCa) have collectively advanced understanding of OHCA.^13^, ^14^, ^15^, ^16^ Furthermore, the ILCOR Research and Registries Committee has played an increasingly important role in global registry collaboration such as reporting OHCA characteristics and outcomes across registries.^2^, ^4^

**Fig. 1:**
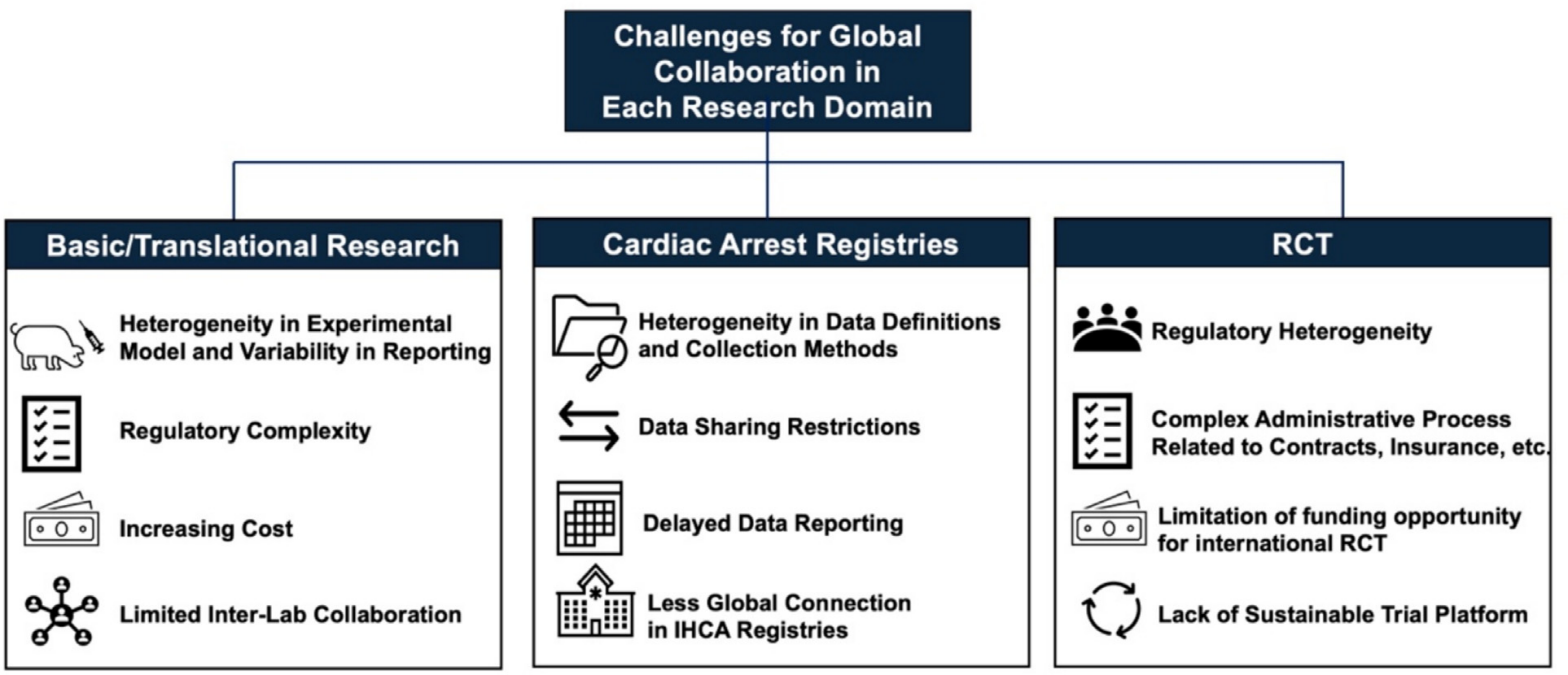
**Challenges in global collaboration of cardiac arrest research**. IHCA, in-hospital cardiac arrest, RCT, Randomized Control Trial.

However, panelists noted several major challenges during the discussion (Fig. 1). First, data ownership and sharing restrictions remain a major impediment. Institutional and national policies often limit secondary data use, and the implementation of data protection laws such as the General Data Protection Regulation (GDPR) in Europe have introduced additional administrative complexity. Negotiating data-use agreements and cross-border governance frameworks consumes substantial time and resources.

Second, heterogeneity in data definitions and collection methods persist despite international efforts, such as the Utstein-style templates developed under ILCOR.^17^ Each registry operates under standardized yet distinct systems of data collection and governance. As one participant observed, “We may all report bystander CPR, but unless the numerator and denominator are harmonized, we are comparing apples and oranges.” This lack of uniformity limits the comparability of results and complicates meta-analyses across regions. Differences in how variables are defined such as return of spontaneous circulation (ROSC), survival to discharge, or neurological outcomes, reduce the ability to perform valid international comparisons. Many participants called for a routine update and harmonization of global consensus on common data elements for both OHCA and IHCA.

Third, delayed data reporting was discussed as a critical limitation. Multinational datasets often rely on retrospective compilation, meaning analyses are based on data that may be several years old and no longer reflective of current systems of care. As one panelist noted, “Four-year-old data cannot answer today’s clinical questions.” Emerging technologies such as federated learning, automated electronic data capture, and cloud-based analytics may provide solutions to these barriers by enabling near real-time data exchange while maintaining local data control.

Fourth, panelists highlighted that IHCA registries such as the Get With The Guidelines–Resuscitation (GWTG-R) program in the United States^18^ and emerging networks in Europe and Asia, are less globally connected, with few platforms enabling multinational comparison. Insufficient implementation of a standardized IHCA data collection format across countries hinders comprehensive analyses of patient characteristics, treatment processes, and outcomes across the globe.

Despite these obstacles, participants expressed optimism about the growing convergence of registry networks. Initiatives such as the Global Out-of-Hospital Cardiac Arrest Registries (GOHCAR) consortium aim to harmonize definitions and facilitate cross-registry collaboration.^9^ Strengthening trust, promoting inclusivity, and standardizing both OHCA and IHCA datasets were viewed as key priorities for the next phase of global cardiac arrest research.

### Basic science and translational research

An important root cause of the lack of progress in improving cardiac arrest outcomes is inadequate discovery and translation of new diagnostic, monitoring, and treatment modalities. Contemporary human cardiac arrest research has largely focused on comparative effectiveness studies evaluating therapeutic strategies already in clinical use. There are few promising new therapies in the translation pipeline.^19^

Underlying causes of the underperforming basic and translational research enterprise are multifactorial. Major contributors include the escalating cost and regulatory complexity of basic and translational cardiac arrest research, challenges in obtaining research funding, declining willingness of high-impact journals to publish experimental cardiac arrest studies, declining numbers of basic scientists and laboratories engaged in cardiac arrest research and limited inter-laboratory collaboration. The ILCOR no longer includes basic and translational research in their evidence evaluation processes. Knowledge gaps published in ILCOR CoSTRs are not rigorously derived and do not address the preclinical literature, further exacerbating the bidirectional translation barrier. The Utstein-Style Guidelines for Uniform Reporting of Laboratory CPR Research have not been updated since publication in 1996 and are rarely cited.^20^, ^21^, ^22^ A recent review of almost 500 cardiac arrest animal studies found significant heterogeneity of the models, variability in definitions and reporting, and many studies with high risk of bias (e.g. lack of adequate randomization and blinding).^23^, ^24^ Taken together, these findings indicate the need to improve the conduct and reporting of animal cardiac arrest research.

The perceived value of basic and translational research has deteriorated in part due the poor track record of translating therapies demonstrated to be effective in preclinical models to positive clinical trials. Contributing to this “failure to translate” is the limited use of adequately powered large animal outcome studies. Also, single-site studies lack the subject and treatment variability inherent in human clinical trials, limiting their reliability in predicting intervention efficacy in human trials. There is also lack of consensus on what preclinical data is needed to justify proceeding to clinical trials or an accepted methodology to determine which interventions are the most promising and should be prioritized for clinical trials. If action is not taken to support and improve the international cardiac arrest basic and translational research infrastructure, we risk losing a key component of the knowledge translation pipeline along with the hope of new discoveries.

#### Clinical trials

Multiple randomized clinical trials have evaluated interventional strategies aimed at improving outcomes after cardiac arrest.^25^ Notable examples from the prehospital setting include the PARAMEDIC trials, which assessed the use of mechanical chest compression devices, the administration of adrenaline during CPR, and the comparison of intraosseous versus intravenous routes for drug delivery.^26^, ^27^, ^28^ An emerging theme across these trials is that, even when a positive direction of effect is observed, the magnitude of the benefit is smaller than initially anticipated. For instance, in the PARAMEDIC 2 trial comparing adrenaline to placebo during CPR, there was a 15% relative increase in the proportion of patients achieving a good functional outcome at 30 days (2.2% vs 1.9%).^28^ However, due to the overall very low survival and good functional outcome rate, the study was underpowered to demonstrate a statistically conclusive effect, resulting in a number needed to treat (NNT) of 333—highlighting the challenge of detecting clinically meaningful effects in a condition with such poor baseline outcomes. In contrast, the effect of adrenaline on return of spontaneous circulation (ROSC) was more pronounced, showing a 15% absolute increase in ROSC rates and an NNT of just 7. On the other hand, the recent trials on double-sequence defibrillation reported absolute improvements in survival in the range of 10–15%, but these findings require replication across different EMS systems and patient populations.^29^

In the field of targeted temperature management, early trials such as the HACA study suggested a substantial benefit, with an NNT of 7.^30^ However, these large treatment effects were not reproduced in later, larger and more methodologically robust trials.^31^, ^32^ In the HYPERION trial, which focused on patients resuscitated from non-shockable rhythms, the absolute difference in good neurological outcome was 4.8% (NNT 21).^33^ Notably, this effect was primarily driven by patients with in-hospital cardiac arrest, where the absolute difference was 10% (NNT 10), compared with only 2% (NNT 50) in OHCA. A Bayesian re-analysis of the available evidence suggests a 76% probability that hypothermia provides some benefit, but the most likely absolute difference in survival is under 2%, corresponding to an NNT of more than 50.^34^ For a devastating condition such as hypoxic–ischaemic brain injury after cardiac arrest, even such modest improvements may be considered worthwhile by many clinicians and patients. What becomes painfully clear, however, is that to reliably detect absolute treatment effects in the range of 2–5%, trials need to be much larger than historically conducted in this field.

### The future of cardiac arrest research: what works well in other successful collaborations

#### Registries

Discussions at the Conference highlighted that the most enduring progress in registry-based cardiac arrest research has come from initiatives grounded in trust, inclusivity, and sustained engagement (Fig. 3). Panelists described how long-standing collaborations such as the CARES in North America and the PAROS have demonstrated the power of using common data structures and regular communication to foster transparency and mutual learning. Their partnership, together with networks like AUS-ROC, ultimately led to the formation of the GOHCAR collaborative—a model for peer-driven progress.^9^ Participants noted that these collaborations were not built through formal mandates but through years of professional relationships, shared goals, and the simple recognition that cardiac arrest outcomes improve when data is shared rather than siloed.

Speakers emphasized that building trust among data custodians remains central to success. For example, panelists shared experiences of initial reluctance from ambulance services to release data until transparent governance and joint authorship policies established confidence in how the information would be used. Similar challenges were seen when reconciling cross-border data protection between the United States and Canada, requiring legal reassurance for automatic transfer of de-identified data. Despite such hurdles, mutual respect and the recognition of shared goals and benefits enabled collaboration to flourish.

Finally, participants highlighted that the sustainability of registry networks depends on continuous communication and inclusivity, including opportunities for emerging young investigators to participate. The strength of these global registries lies not only in harmonized data but in the relationships and trust that allow them to persist across regions and decades.

### Basic science and translational research

We envision a future state in which international collaboration is a major driving force behind laboratory science in the field of cardiac arrest resuscitation. Similar to the way clinical registries and clinical trial networks have been foundational in advancing clinical cardiac arrest research, preclinical registries and preclinical trial networks could have an even more profound impact on the advancement of basic and translational research, especially if done at the international level. This infrastructure would also provide a platform for international exchange of basic science and translational research trainees that would nurture a sustainable pipeline of well-trained future resuscitation scientists and a culture of international collaboration.

**Fig. 2:**
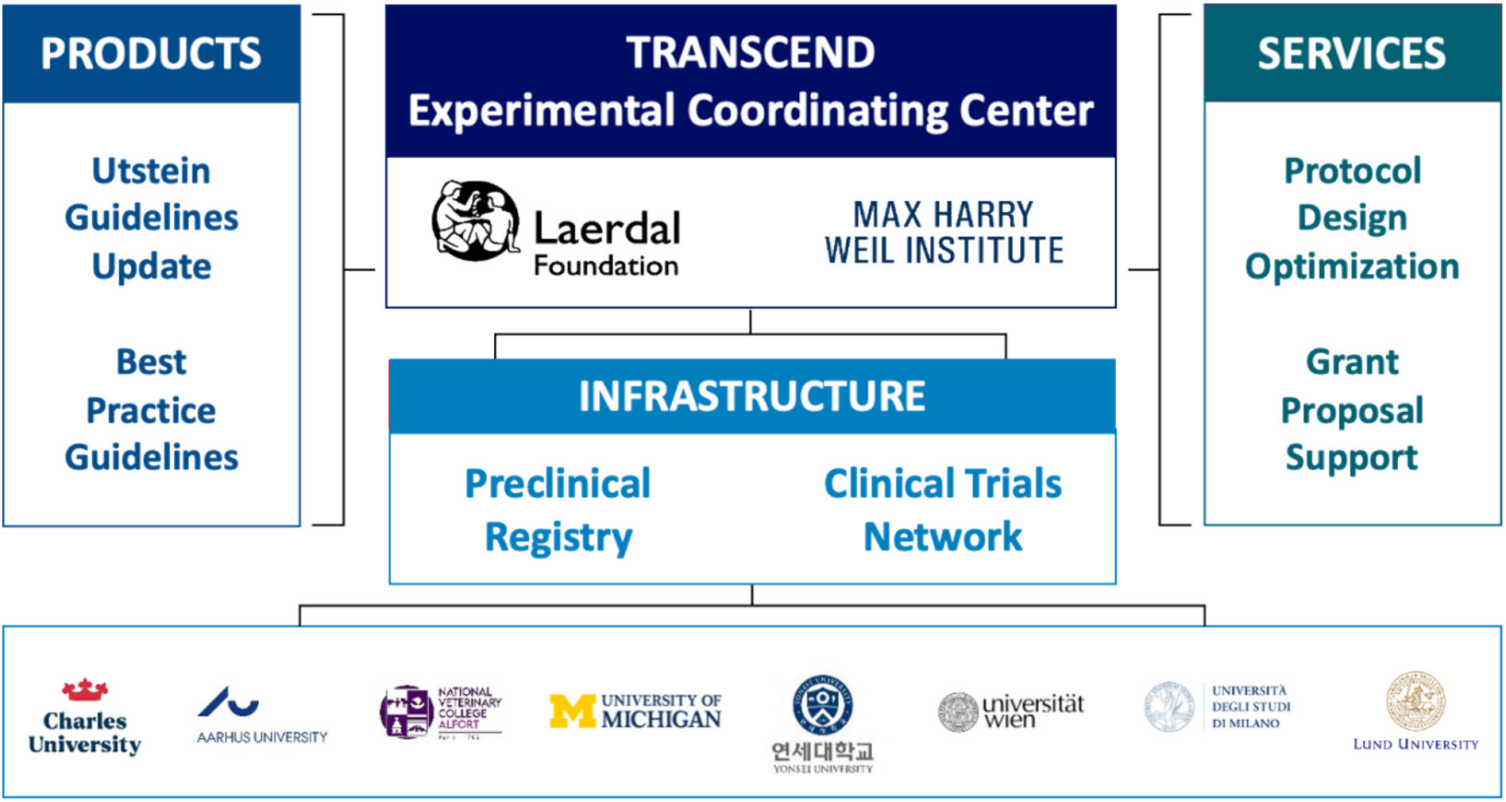
**The TRANSCEND programmatic structure and participating institutions**.

The Transcontinental Cardiac Arrest Experimental Network for Discovery (TRANSCEND), funded by the Laerdal Foundation, was recently created to help achieve the above stated vision (Fig. 2). The long-term objective of TRANSCEND is to maximize the discovery and translation of new therapies for cardiac arrest by creating an international program that will advance the quality, effectiveness, and sustainability of the preclinical cardiac arrest research enterprise. The program strives to foster an international community of laboratory scientists with a common mission and create an infrastructure that aligns incentives, removes barriers, and supports the next generation of resuscitation scientists. Success will be measured by the pipeline of new cardiac arrest therapies catalyzed by the program. Key deliverables will include: (1) An Utstein-style conference and publication of updated standards for animal cardiac arrest experimental designs and outcomes in partnership with the ILCOR, and (2) creation of the TRANSCEND Experimental Coordinating Center (ECC). The TRANSCEND ECC will provide infrastructure to support a preclinical registry, create a preclinical trial research network, provide experimental protocol design and grant proposal support, and publish systematic reviews and best practices for cardiac arrest experimental protocols and assays. The ECC will also serve a resource for investigators and industry seeking to perform multicenter preclinical trials using TRANSCEND affiliated labs. This will include use of the preclinical registry for preliminary data and sample size calculations, experimental protocol optimization, grant proposal coordination to fund international multicenter studies, data collection and analysis using our established registry platform. These resources will catalyze the discovery and translation of new therapies in a way that is more likely to result in positive clinical trials, sustain individual cardiac arrest laboratories, and support the next generation of experimental resuscitation scientists.

**Fig. 3:**
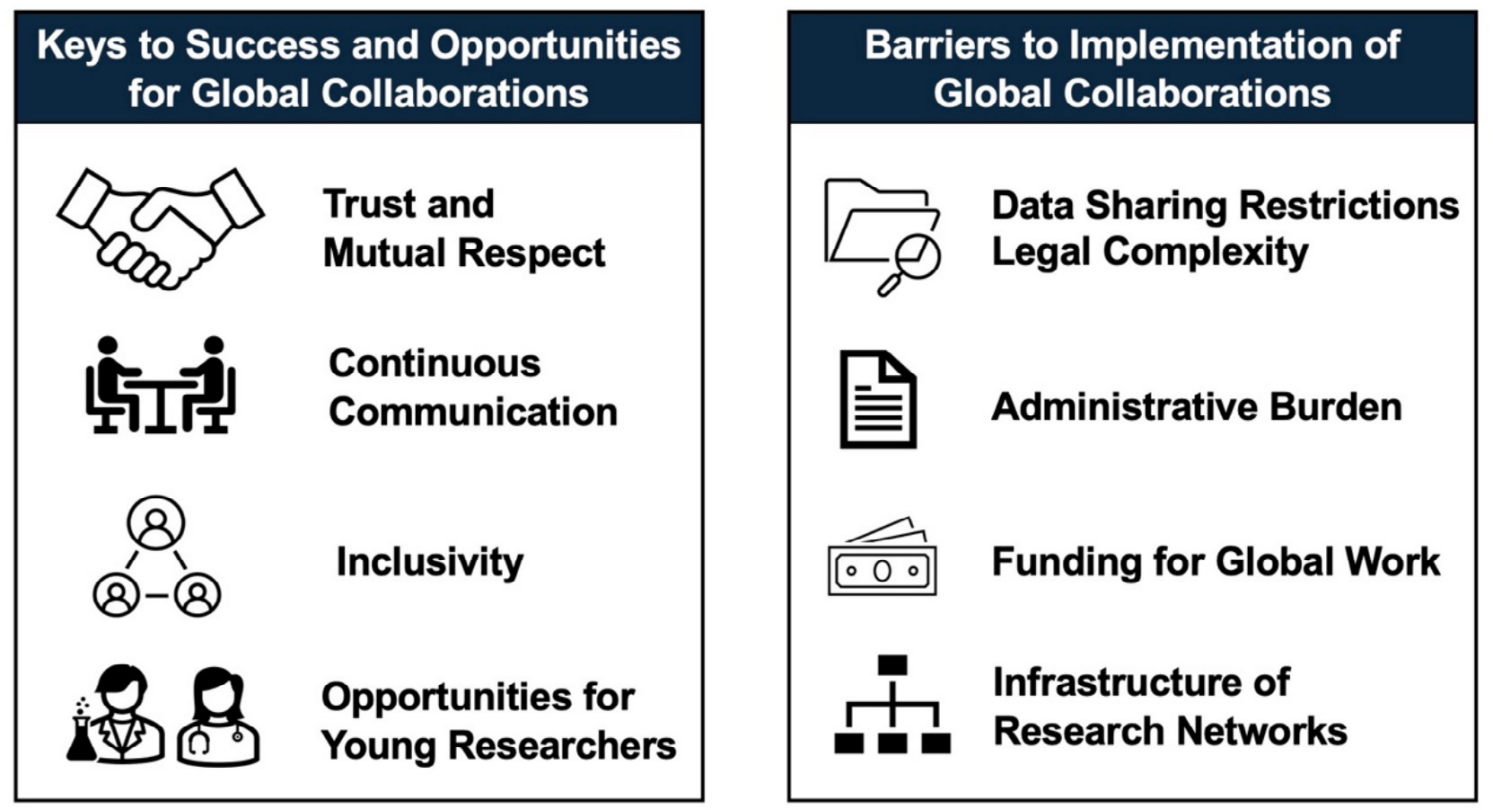
**Keys to success and barriers for global collaborations**.

If successful, TRANSCEND could become an indispensable catalyst for the translation of new cardiac arrest therapies to clinical trials by creating the infrastructure needed to perform multicenter, prospective, double-blind, randomized preclinical trials that reliably replicate the human condition and predict human cardiac arrest trial results. An envisioned future state would include a robust pipeline of new cardiac arrest therapies shown to be effective in rigorous, unbiased multicenter preclinical trials that matches or exceeds the capacity to perform the subsequent clinical trials. The productivity and efficacy of our knowledge translation enterprise could be significantly improved, ultimately leading to more effective treatments that improve patient outcomes. Transition from Laerdal startup funding to an independently sustainable program will likely require government, philanthropic, foundation, or industry support.

#### Clinical trials

The ongoing Sedation, TEmperature and Pressure after Cardiac Arrest and REsuscitation (STEP CARE), which has now recruited nearly 75% of its planned population, exemplifies a shift toward larger-scale collaborative research.^35^, ^36^, ^37^, ^38^ Other ongoing large-scale studies include the AIRWAYS-3 and ICECAP studies.^39^, ^40^ The ICECAP trial compares different strategies for mean arterial pressure, sedation depth, and fever control in comatose post–cardiac arrest patients treated in the ICU, and is powered to detect absolute differences of less than 6% in neurological outcomes. To achieve this, STEP CARE is running at more than 60 ICUs across Europe, the Middle East, Asia, Australia, and New Zealand. It is becoming increasingly evident that international collaboration is essential to enable trials enrolling 4000–5000 patients—numbers that are routine in the field of acute cardiovascular care, such as trials of patients with acute coronary syndromes.^41^ One particularly appealing future direction is the establishment of platform trials with continuous recruitment and the ability to test multiple interventions within a shared infrastructure.^42^ These trials could run over many years, allowing adaptive randomization and efficient evaluation of several therapeutic strategies in parallel. Recent experiences from multinational critical care research networks have demonstrated that such efforts are feasible. If sustained, they may pave the way for a truly evidence-based approach to managing patients during and after cardiac arrest.

### Current gaps and barriers to implementation

During the discussion, several persistent barriers and potential enablers for effective international collaboration in cardiac arrest research were highlighted (Fig. 3). Data sharing and legal complexity emerged as a recurring theme. Participants noted that cross-border data transfer remains challenging due to varying national regulations and institutional risk management policies. In Europe, the General Data Protection Regulation (GDPR) was cited as a frequent obstacle creating unnecessary hurdles for collaborative data analysis. Similar issues have arisen in data exchanges between the United States and Canada. Despite these challenges, some progress has been achieved through legal reforms such as Article 35 of the EU Clinical Trials Regulation, which now allows emergency trials to proceed with deferred or opt-out consent, offering a more flexible framework for cardiac arrest research in Europe.

Another area of concern was the substantial administrative burden associated with multicenter studies. Panelists described the extensive time and effort required to negotiate contracts, insurance, and authorship agreements across multiple jurisdictions, often driven by overly cautious legal and institutional review processes. In one example, significant insurance expenditures were incurred solely to meet differing national requirements. Such bureaucratic demands were viewed as diverting valuable resources and focus away from scientific activity.

Additionally, funding was identified as one of the most difficult barriers to overcome. Current funding mechanisms are primarily national, leaving little room for multinational initiatives. Participants discussed the need for an international consortium of laboratories capable of conducting multicenter preclinical trials, supported initially by international foundations or industry partners to demonstrate feasibility and attract government funding.

Finally, concerns were raised regarding infrastructure and sustainability. Many large trials build extensive collaborative frameworks that are dismantled once a study concludes. The group emphasized the importance of maintaining ongoing platforms that can continuously address new research questions, similar to the adaptive model seen in REMAP-CAP during the COVID-19 pandemic.^43^

### Priorities for implementation

#### Building international research networks

During the panel discussion, participants emphasized that the next phase of cardiac arrest research must focus on building sustainable international research networks and platforms. Such networks have the potential to overcome persistent barriers in data sharing, standardization, and funding, while also amplifying the global impact of resuscitation science and implementation. One of the lessons from recent years is the potential of clinical trial networks and large collaborative registries to deliver answers that single centers or even single countries cannot achieve alone. Networks such as the STEPCARE trial group, CARES in the United States, the Resuscitation Outcomes Consortium (ROC) in North America, EuReCa in Europe, the PAROS in Asia, the Aus-ROC in Australian and New Zealand, and the GOHCAR, were highlighted as successful models that demonstrate the power of shared infrastructure.^9^, ^13^, ^14^, ^16^, ^44^, ^45^ By pooling resources and patient populations, these collaborations have been able to overcome data scarcity, harmonize definitions, and generate findings that are widely generalizable. Moreover, the existence of such networks facilitates access to funding, as funders are often more willing to support large, coordinated initiatives with broad reach. Several speakers also mentioned the importance of inclusive governance, transparency, and equitable participation**,** which were seen as prerequisites for sustaining long-term engagement.

Another theme raised during the discussion was the need to embed research more deeply into routine clinical care through robust digital infrastructure and continuous data collection. This integration, as illustrated by networks such as ROC, was described as essential to maintaining data quality and real-time feedback.

Panelists also agreed that similar strategies should extend to preclinical research**,** where coordinated basic science and translational research networks based on updated Utstein-style guidelines could enhance reproducibility and accelerate translation. The newly established TRANSCEND network was highlighted as a model initiative that embodies these principles.

Finally, participants stressed the importance of investing in the next generation of resuscitation researchers**.** Opportunities such as exchange visits, joint fellowships, and structured mentorship were viewed as critical to sustaining the collaborative culture necessary for future progress.

### Call to action

In concluding discussions, panelists emphasized that strengthening international research networks is essential given the global burden of time-critical conditions**.** Each year, more than 30 million people die from causes such as cardiac arrest, trauma, stroke, sepsis, and maternal emergencies—most of which are preventable through timely recognition, rapid intervention, and coordinated systems of care.^46^, ^47^, ^48^ Cardiac arrest research therefore occupies a critical interface within the broader chain of survival, which is applicable across these time-critical conditions. Implementing such strategies, particularly in low- and middle-income countries, offers substantial potential to reduce preventable mortality.

## Conclusion

The priority for the coming decade is clear: resuscitation science should play a greater role in saving lives globally. Building and sustaining international research networks and platforms should be practical pathways to achieving this goal, delivering robust, generalizable evidence and implementing better clinical practice worldwide. At the same time, expanding the scope of resuscitation research beyond high-income countries and beyond cardiac arrest alone acknowledges the broader potential of resuscitation science to reduce millions of preventable deaths each year.

## Ethical approval

Not applicable.

## Consent for publication

Not applicable.

## Availability of data and materials

Not applicable.

## CRediT authorship contribution statement

**Yohei Okada:** Writing – review & editing, Writing – original draft, Conceptualization. **Janet E. Bray:** Writing – review & editing, Writing – original draft, Conceptualization. **Robert W. Neumar:** Writing – review & editing, Writing – original draft, Resources, Funding acquisition, Conceptualization. **Bryan F. McNally:** Writing – review & editing, Writing – original draft, Conceptualization. **Markus B. Skrifvars:** Writing – review & editing, Writing – original draft, Conceptualization. **Laurie J. Morrison:** Writing – review & editing, Writing – original draft, Conceptualization. **Niklas Nielsen:** Writing – review & editing, Writing – original draft, Conceptualization. **Theresa Olasveengen:** Writing – review & editing, Writing – original draft, Conceptualization. **Marcus E.H. Ong:** Writing – review & editing, Writing – original draft, Supervision, Conceptualization.

## Funding

Funding for the conference was provided by the Max Harry Weil Institute for Critical Care Research and Innovation and corporate sponsors including ZOLL Medical, Stryker, Corpuls, Resuscitec, Phillips, Shiller Medical, the American Heart Association, add the American Red Cross.

## Declaration of competing interest

YO received a research grant from the ZOLL Foundation. MO reports grants from the Laerdal Foundation, Laerdal Medical, and Ramsey Social Justice Foundation for funding of the Pan-Asian Resuscitation Outcomes Study an advisory relationship with Global Healthcare SG, a commercial entity that manufactures cooling devices; and funding from Laerdal Medical on an observation program to their Community CPR Training Center Research Program in Norway. MO is a Scientific Advisor to TIIM Healthcare SG and Global Healthcare SG. JB reports grants from the Laerdal Foundation and Heart Foundation of Australia. RWN is immediate past chair of the International Liaison Committee on Resuscitation (ILCOR) and director of TRANSCEND. He reports research grant funding from the National Institutes of Health, American Heart Association, and Laerdal Foundation, and research equipment support from BrainCool, Corpuls, and Nonin. These organizations had no role in the writing of this review. LJM is a member of ILCOR. She holds research grants from Heart and Stroke Canada and the Canadian Institute for Health Research. JB is an Associate Editor, RWN a Special Issue Editor and TO a member of the Editorial Board of Resuscitation Plus. JB, RWN, TO, MBS and MO are members of the Editorial Board of Resuscitation.
